# Sexual behavior and factors associated with young age at first intercourse and HPV vaccine uptake among young women in Germany: implications for HPV vaccination policies

**DOI:** 10.1186/1471-2458-14-1248

**Published:** 2014-12-05

**Authors:** Cornelius Remschmidt, Michaela Fesenfeld, Andreas M Kaufmann, Yvonne Deleré

**Affiliations:** Department for Infectious Disease Epidemiology, Immunization Unit, Robert Koch Institute, Seestrasse 10, 13353 Berlin, Germany; Clinic for Gynaecology, Charité-Universitätsmedizin Berlin, Berlin, Germany

**Keywords:** Sexual behavior, First intercourse, Sexually transmitted infections, HPV vaccination

## Abstract

**Background:**

In Germany, immunization against human papillomaviruses (HPV) is free of charge for all females aged 12 to 17 years. Since HPV infection rates rise soon after first intercourse, immunization against HPV should be completed before sexual debut. Knowledge of country-specific data on age at first intercourse and related risk factors is important to optimize prevention of HPV and other sexually transmitted infections. Therefore, the primary aim of this study was to describe sexual behavior in young women in Germany. Secondary aims were to identify factors that are (i) associated with younger age at first intercourse and (ii) with HPV vaccine uptake.

**Methods:**

Between 2010 and 2012, we conducted a cross-sectional study among randomly selected women aged 20 to 25 years in Germany. We used a structured, self-administered questionnaire to collect sociodemographic data, information on sexual habits such as age at first intercourse, and information on HPV vaccine uptake. We used univariate and multivariate logistic regression analyses to identify factors associated with younger age at first intercourse and with HPV vaccine uptake.

**Results:**

A total of 823 women (response rate: 14.2%) participated, 785 (95.4%) of which reported having had intercourse already. 70% of these women experienced first intercourse before the age of 18 years. However, less than 5% were younger than 14 years at sexual debut. Younger age at first intercourse was independently associated with a higher number of sexual partners, smoking, and past pregnancies. HPV vaccine uptake was associated with higher education, whereas smoking and a migrant background reduced the chance of being vaccinated.

**Conclusion:**

In Germany, only a small proportion of women experienced first intercourse before the age of 14 years. Younger age at first intercourse was associated with behavior that might increase the risk of HPV infections or other sexually transmitted infections. Therefore, to optimize the HPV vaccination strategy, HPV vaccination series should be completed before the age of 14 years in Germany.

## Background

Human papillomaviruses (HPV) are the most common sexually transmitted viruses [1], with a life-time risk of cervical infection of up to 80% in the female population [2]. Two safe and effective vaccines covering high-risk HPV types 16 and 18 are licensed. Since HPV infection rates rise soon after first intercourse [3, 4] and since HPV vaccines effectiveness is reduced in infected individuals [5], vaccination series should be completed before sexual debut. In Germany, the Standing Committee on Vaccination (STIKO) recommends HPV vaccination free of charge for all females aged 12 to 17 years since 2007 [6]. Both, the quadrivalent and the bivalent vaccine are available in Germany.

Several risk factors for HPV transmission have been identified so far, with high-risk sexual behavior, such as younger age at first sexual intercourse [1, 7] and higher numbers of present or recent sexual partners [8–10] being the most important ones. However, few studies have analyzed sexual behavior in Germany and - to the best of our knowledge - no study has assessed factors associated with younger age at first intercourse in Germany so far.

Since national data on sexual behavior are important to optimize prevention strategies against sexually transmitted infections, the primary aim of this study was to describe sexual behavior in young women in Germany. Secondary aims were to identify factors that are associated (i) with younger age at first intercourse and (ii) with HPV vaccine uptake.

## Methods

### Study design and study population

This study was nested in a study designed to determine HPV prevalence in Germany via home-based self-sampling [11]. Briefly, we conducted a cross-sectional study among 20 to 25 year old women in Germany between 2010 and 2012. We used a two-step cluster sampling approach based on the protocol of the German Health Interview and Examination Survey for Adults [12]: Within selected sampling points women were randomly selected from local registration offices. An invitation letter, a self-sampling kit, and a questionnaire were mailed to each woman who gave written informed consent. Self-sampling was performed by cervicovaginal lavage (5 ml volume) with the first generation Delphi-Screener (Delphi-Bioscience, Scherpenzeel, The Netherlands) as described previously [13]. The study was approved by the local ethics committee (Charité, EA2/028/10) and registered at Deutsches Register Klinischer Studien (DRKS 00000599).

### Questionnaire

With a structured, self-administered questionnaire in the German language we obtained information on sociodemographic factors, data on sexual behavior (such as age at first intercourse and number of lifetime and recent (i.e. in the previous 12 months) sexual partners), medical and smoking history, contraceptive use, past pregnancies, past delivery of a child, as well as HPV-vaccine uptake (time of vaccination and number of doses). The questionnaire has been developed by the Robert Koch Institute and was previously used in another study [14]. Details about the questionnaire and definitions are described elsewhere [11]. Data from the questionnaires were self-reported and not validated.

### Statistical analysis

We calculated proportions on young age at first intercourse, contraceptive use and past pregnancies with corresponding 95% confidence intervals (95% CI). We evaluated the lifetime number of sexual partners by estimating the median, range and interquartile bounds (IQB). For comparison of proportions of categorical variables, we used chi-squared test and Fisher’s exact test, and Student’s t-test for numerical variables. Differences between three age groups (20–21, 22–23 and 24–25 years) were assessed by the Kruskal-Wallis-test for categorical variables and by analysis of variance (ANOVA) for continuous variables.

The age cut-off for â€˜young’ age at first intercourse was defined as the lower quartile (age ≤ 14) and a â€˜high’ number of lifetime sexual partners was defined as the upper quartile (≥ 7 partners) in the respective variable distribution. For assessing factors associated with HPV vaccine uptake, women with at least one HPV vaccine dose were defined as “vaccinated”.

In order to identify factors associated with (i) young age at first intercourse and (ii) HPV vaccine uptake, we conducted univariate analyses for all binary or categorical exposure variables and calculated odds ratios (OR) and 95% confidence intervals (95% CI). We then used multivariate logistic regression analysis and included variables with a p-value <0.1 in univariate analysis. In addition, we included important potential confounding factors on the basis of the current literature. Two-sided hypothesis tests were performed and a p-value of less than 0.05 was considered as statistically significant. Missing data were treated as such and not imputed. We used the statistical software package STATA, version 11 (STATA Corp., College Station, TX, USA).

### Representativeness of study population

We used data of the German Federal Statistical Office (as of 31 December 2011 [15]) to compare characteristics of our study population with the general German female population of the same age.

## Results

### Overall study population

**Figure 1:**
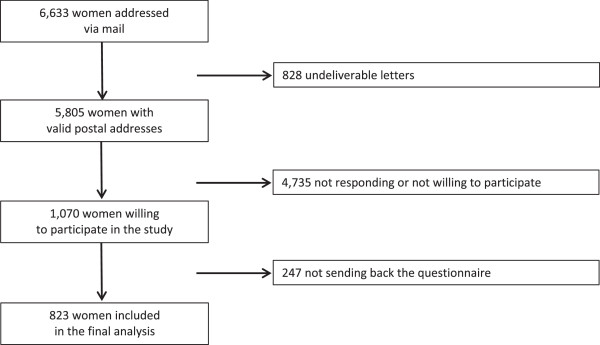
**Flow chart of the study.**

Overall, 823 out of 5805 women with a valid postal address (14.2%) completed the questionnaire and were included in the analysis (Figure 1). Mean age of participants was 22.6 years (standard deviation (SD) = 1.6). Key characteristics of the study population compared to the general female population in Germany of the same age group are presented in Table 1.

**Table 1:** **Baseline characteristics of the study population, Germany 2010–2012**

	Study population (n = 823)	General female population aged 20–24 years, Germany
	% (95% CI)	%
**Residency**		
Western federal states	82.5 (79.7-85.0)	83.7^1^
Eastern federal states	18.3 (17.5-20.3)	16.3^1^
**Living in a city with**		
< 500,000 inhabitants	83.0 (80.0-85.6)	80.5
≥ 500,000 inhabitants	16.9 (14.4-19.7)	19.5
**Current smoker**		
No	69.9 (66.6-73.0)	67.2
Yes	30.1 (26.9-33.4)	32.8
**Migrant background**		
No	85.9 (83.3-88.2)	78.0
Yes	14.1 (11.8-16.7)	22.0
**Educational status**		
Low	5.8 (4.3-7.6)	22.2
Medium	28.6 (25.5-31.8)	32.8
High	65.6 (62.3-68.9)	44.9

^1^comparison group: women aged 20–25 years.

*95*% *CI*, 95% confidence interval.

### Sexual behavior

Overall, 95.4% (95% CI, 93.7-96.7%) had experienced sexual debut at the time of the study. More than half of the women were 16 years or older at sexual debut, whereas only 4.6% (95% CI, 3.3-6.3%) were younger than 14 years (Table 2). 73.5% (95% CI, 70.3-76.5%) were living in a relationship and 69.6% (95% CI, 66.5-72.8%) reported having had just one sexual partner in the previous 12 months. The proportion of women with more than one sexual partner in the last 12 months was significantly higher among women not living in a relationship as compared to those living in a relationship (35.6% vs. 16.3%, p-value < 0.01).

**Table 2:** **Sexual behavior of the study population, Germany 2010-2012**

	Proportion of women (n = 823)	
Variable/Category	n/N	% (95% CI)
**Living in a partnership**	604/822	73.5 (70.3-76.5)
**Age at first intercourse (years)**		
No sexual intercourse	38/823	4.6 (3.2-6.1)
< 13	8/823	1.0 (0.3-1.6)
13	30/823	3.6 (2.4-4.9)
14	104/823	12.6 (10.4-14.9)
15	155/823	18.8 (16.2-21.5)
16	149/823	18.1 (15.5-20.7)
17	125/823	15.2 (12.7-17.6)
≥18	214/823	26.0 (23.0-29.0)
**Lifetime no. of sexual partners**		
0	38/819	4.6 (3.2-6.1)
1	265/819	19.6 (16.9-22.4)
2-3	264/819	24.4 (21.5-27.4)
4-6	169/819	26.2 (23.2-29.3)
≥ 7	83/819	25.0 (22.1-28.0)
**Median lifetime no. of partners** (IQB)	819	4 (2–7)
Mean (SD; range)		5.2 (6.1;0–80)
**Recent sexual partners** ^**1**^		
0	72/820	8.8 (6.8-10.7)
1	571/820	69.6 (66.5-72.8)
≥ 2	177/820	21.6 (18.8-24.4)
**Bisexual experience**	37/786	4.7 (3.2-6.2)
**Any contraceptive use (ever)**	763/823	92.7 (90.1-94.5)
**Contraceptive method**		
Pill (ever)	734/823	89.2 (87.1-91.3)
Pill (current use^**1**^)	416/823	50.6 (47.1-54.0)
Condoms (ever)	568/823	69.0 (65.8-72.2)
Condom use during ONS	660/817	80.8 (77.9-83.4)
Vaginal ring (ever)	89/823	10.8 (8.7-12.9)
Implant (ever)	12/823	1.5 (0.6-2.3)
**History of genital warts**	19/801	2.4 (1.3-3.4)
**Ever pregnant**	70/821	8.5 (6.6-10.4)
**Delivery of a child**	46/817	5.6 (4.0-7.2)

*IQB* interquartile bounds; *SD* standard deviation; *95*% *CI* 95% confidence.

Interval; ^1^ in the past 12 months.

### Factors associated with young age at first intercourse

17.2% (95% CI, 14.7-20.0%) of sexually active women reported that they experienced first intercourse before or at age of 14 years (Table 2). More than half of the women were older than 15 years at sexual debut and one fourth was older than 17 years. In univariate analysis younger age at first intercourse (defined as ≤ 14 years) was independently associated with lower educational status, current smoking, a higher number of recent and lifetime sexual partners, bisexual experience, usage of contraceptive pill, past pregnancy, and genital warts (data not shown).

The final multivariate model indicated that current smoking, a higher number of sexual partners, and past pregnancy were independently associated with young age at first intercourse (Table 3). In addition, women with younger age at sexual debut were more likely to have bisexual experience and to have used the contraceptive pill. Adjusting for age did not substantially affect the model.

**Table 3:** **Adjusted odds ratio (OR) and 95**% **confidence intervals for factors associated with young age at first intercourse (defined as ≤ 14 years), Germany 2010-2012**

Variable and category	Age at first intercourse ≤ 14 vs. > 14 years			
	Adjusted OR			
	n	% pos	(95% CI)	p
**Current smoker**				
No	539	11.7	1	-
Yes	244	32.4	2.3 (1.5-3.6)	<0.01
**Educational status**				
Low	46	41.3	1	-
Medium	225	24.0	0.5 (0.2-1.1)	0.08
High	510	13.3	0.3 (0.2-0.7)	0.01
**Lifetime no. of sexual partners**				
1	161	4.3	1	-
2-3	200	11.5	2.5 (1.0-6.5)	0.05
4-6	215	20.8	4.6 (1.9-11.2)	<0.01
≥ 7	205	30.2	5.0 (2.0-12.4)	<0.01
**Bisexual expierience**				
No	738	16.1	1	-
Yes	37	51.3	3.2 (1.5-6.8)	<0.01
**Contraceptive pill (ever)**				
No	62	9.7	1	
Yes	723	18.9	2.7 (1.0-7.0)	0.05
**Past pregnancy**				
No	714	15.5	1	-
Yes	70	44.3	3.0 (1.6-5.4)	<0.01

*OR* odds ratio; *95*% *CI* 95% confidence interval.

### Contraception and pregnancy

A total of 92.7% (95% CI, 90.1-94.5%) women reported usage of any type of contraception, with the contraceptive pill and condoms being the most common ones (Table 2). 10.8% (95% CI, 8.7-12.9%) of the participants had ever used a vaginal ring and 1.5% (95% CI, 0.6-2.3%) an implant. Overall, 80.8% (95% CI, 77.9-83.4%) of women stated that they would always use condoms during a one-night stand. However, the probability of consistent condom use decreased with an increasing number of a woman's lifetime sexual partners (p-value <0.01). The proportion of women with a previous pregnancy and who delivered a child was 8.6% (95% CI, 6.6-10.4%) and 5.6% (95% CI, 4.0-7.2%), respectively.

### HPV vaccine uptake

Two hundred thirty-four out of 772 participants (30.3%, 95% CI, 27.1-33.7%) with information on HPV vaccination status received at least one dose of an HPV vaccine and 206 (26.7%, 95% CI, 23.6-29.9%) received the full course. Statistically, vaccinated women were significantly younger than unvaccinated women (mean age, 21.5 years (SD = 1.5) vs. 23.0 years, (SD = 1.4), p-value <0.01). Fifty-six (30.1%, 95% CI, 23.6-37.2%) out of 186 women received the first dose of vaccination before their first intercourse. Nineteen (3.5%, 95%, 2.1-5.5%) out of 538 unvaccinated women reported that they were still planning to receive the HPV vaccine. All of these women, except one, already had had their sexual debut.

In multivariate analysis HPV vaccine uptake was independently associated with younger age and a higher educational status. Vice versa, active smoking and migrant background were associated with a lower chance of being vaccinated (Table 4).

**Table 4:** **Adjusted odds ratio (OR) and 95**% **confidence intervals for factors associated with HPV-vaccine uptake, Germany 2010–2012**

Variable and category	n	% with≥1 HPV vaccine dose	Adjusted OR (95% CI)	p-value
**Age (years)**	758	-	0.5 (0.4-0.6)	< 0.01
**Current smoker**				
No	547	35.5	1	-
Yes	223	17.9	0.4 (0.3-0.7)	<0.01
**Migrant background**				
No	657	32.9	1	-
Yes	108	13.0	0.4 (0.2-0.8)	<0.01
**Educational status**				
Low	37	10.8	1	-
Medium	219	27.4	3.8 (1.2-12.3)	0.03
High	511	33.1	4.7 (1.5-14.8)	<0.01

*OR*odds ratio; *95*% *CI* 95% confidence interval.

## Discussion

In this study, more than 70% of women experienced their first intercourse before age of 17, but less than 5% before age of 14. Factors strongly associated with younger age at first intercourse were a high number (≥ 7) of sexual partners during lifetime, current smoking and a past pregnancy. HPV vaccine uptake was higher in women with higher educational status, whereas uptake was lower in smokers and in women with a migrant background.

National data about sexual behavior in young adults is scarce in Germany. To optimize prevention strategies against sexually transmitted infections, these data is urgently needed. Our results on age at first intercourse are comparable with data obtained in other studies: Crochard et al. reported results from seven European countries where the majority of young women experienced sexual debut between 15 and 18 years (1st to 3rd quartile); less than 5% of girls in this study (4.6% in our study) were younger than 14 years [16]. In a large population-based study in four Nordic countries conducted 2004–2005, Jensen and colleagues found that 2.7% ─ 4.7% of women aged 18 to 25 years had experienced sexual debut at, or before age of 13 years, and 10.6% ─ 16% before the age of 15, respectively [17]. Finally, a representative survey on youth sexuality among 14 to 17-year-olds in Germany in 2009 revealed that 66% of girls have had first sexual intercourse before the age of 18 (74% in our study) and 7% before the age of 15 (17% in our study) [18].

The use of contraceptives was very common in our study population, with the contraceptive pill and condoms being the most popular ones. 80% of participants reported that they would use condoms regularly during one-night stands. These findings might be a positive effect of an ongoing public campaign about condom use and prevention of sexually transmitted infections in Germany [19]. However, the probability of consistent condom use decreased with an increasing number of a woman's lifetime sexual partners. Regarding prevention strategies against sexually transmitted infections, benefits of consistent condom use needs to be addressed continuously towards young adults. Since condom use may imply a lesser protection against HPV infection than against several other sexually transmitted infections, [20, 21] HPV vaccination needs to be promoted as well.

The fact that younger age at first intercourse is associated with risky (sexual) behavior is corroborated by other studies that have shown that early intercourse is associated with higher numbers of sexual partners, bisexual experience, smoking and early pregnancy later in life [22–25]. In addition, women with a higher number of sexual partners [17] also had less consistent condom use, which in turn increased the risk of acquiring sexual transmitted infections [23, 24], including HPV. Several large population-based studies [8, 9, 11, 26] found that a higher number of sexual partners independently increased the risk for HPV infection and, in consequence, increased the risk of pre-cancer and cancer lesions [27]. To this end, women with younger age at first intercourse are at particularly high risk for HPV infection and have the greatest need of getting vaccinated at younger age.

In line with current literature, HPV vaccine uptake was associated with higher education [28–31]. Interestingly, women with a migrant background had a lower chance of being vaccinated. A Canadian study assessing awareness, knowledge and attitudes towards the HPV vaccines found that awareness was higher in Caucasian compared to non-Caucasian women [32]. If the effect in our study is real and not affected by participation bias, efforts are needed to understand possible barriers against HPV vaccination in women with migrant background and to increase vaccine-uptake in this population. However, when factors associated with HPV vaccination are interpreted, the age of study participants (with regard to sexual debut) and endorsement date of the recommendation for HPV vaccination have to be taken into account. Women aged 25 years at the time of our study were 20–22 years old when HPV vaccination was adopted into the national immunization schedule in Germany. Therefore, it is not surprising that a large proportion of the women received the vaccine after sexual debut.

The findings of our survey have important implications for the target population for HPV vaccination in Germany: since rates of HPV rise soon after first intercourse and since HPV vaccination is less effective in infected individuals [3, 4], HPV vaccination is recommended to be administered before sexual intercourse [33]. According to our data, HPV vaccination series in Germany should be completed before the age of 14 years, when the majority of girls had not experienced sexual debut. Physicians should be aware of these data when discussing the optimal time point of the HPV vaccination with their young patients and their parents. Finally, it should be considered, whether the HPV vaccination strategy could be optimized by preponing the recommended age at which HPV vaccination should be initiated.

### Limitations

Some limitations of our study need to be taken into account. First, the response rate was low (14%) with the potential of introducing participation bias. Since this study was implemented in a population based survey to evaluate HPV prevalence using a self-sampling method, the response rate was comparable to a similar study [34]. However, women with a migrant background were underrepresented and those with a higher education were overrepresented. As educational status was independently associated with young age at first intercourse, it is possible that the true proportion of these women is higher than estimated by our study. However, other characteristics of our study sample, such as place of residency, living in a city, and smoking behavior were comparable to the German female population of similar age groups. Second, information on sexual behavior and vaccination status were self-reported and could be subject to misclassification. Third, due to the sensitive nature of the topic on sexual behavior, social desirability bias cannot be excluded. Although anonymity and confidentiality was ensured, women might have had the tendency to give more “desirable” answers with for example a lower number of sexual lifetime partners or an older age at first intercourse. Forth, a long time interval since relevant exposures (e.g., time at first intercourse) may have led to miscalculation by participants and may have influenced the magnitude of relative risk estimates.

## Conclusion

Although hampered by its low response rate, our study indicate that in Germany the majority of young women experienced first sexual intercourse before the age of 18 years, but only a small proportion before the age of 14 years. Usage of the contraceptive pill and condoms was common in our study population. However, condom use may imply lesser protection against HPV than against other sexually transmitted infections, therefore HPV vaccination needs to be promoted as well. Since younger age at first intercourse was associated with behavior that might increase the risk of HPV infections, HPV vaccination series should be completed before the age of 14 years in Germany.

## Abbreviations

CI: Confidence Interval
DRKS: German Clinical Trials Register [Deutsches Register Klinischer Studien]
HPV: Human Papillomavirus
OR: Odds Ratio
RKI: Robert Koch Institute
STIKO: Standing Committee on Vaccination.

## References

[1] Veldhuijzen NJ, Snijders PJ, Reiss P, Meijer CJ, van de Wijgert JH (2010). Factors affecting transmission of mucosal human papillomavirus. Lancet Infect Dis.

[2] Syrjanen K, Hakama M, Saarikoski S, Vayrynen M, Yliskoski M, Syrjanen S, Kataja V, Castren O (1990). Prevalence, incidence, and estimated life-time risk of cervical human papillomavirus infections in a nonselected Finnish female population. Sex Transm Dis.

[3] Winer RL, Lee SK, Hughes JP, Adam DE, Kiviat NB, Koutsky LA (2003). Genital human papillomavirus infection: incidence and risk factors in a cohort of female university students. Am J Epidemiol.

[4] Collins S, Mazloomzadeh S, Winter H, Blomfield P, Bailey A, Young LS, Woodman CB (2002). High incidence of cervical human papillomavirus infection in women during their first sexual relationship. BJOG.

[5] Group FIS (2007). Prophylactic efficacy of a quadrivalent human papillomavirus (HPV) vaccine in women with virological evidence of HPV infection. J Infect Dis.

[6] Robert Koch Institute (2007). Impfung gegen humane Papillomaviren (HPV) für Mädchen von 12 bis 17 Jahren – Empfehlung und Begründung. Epidemiologisches Bull.

[7] Roset Bahmanyar E, Paavonen J, Naud P, Salmeron J, Chow SN, Apter D, Kitchener H, Castellsague X, Teixeira JC, Skinner SR, Jaisamrarn U, Limson GA, Garland SM, Szarewski A, Romanowski B, Aoki F, Schwarz TF, Poppe WA, De Carvalho NS, Harper DM, Bosch FX, Raillard A, Descamps D, Struyf F, Lehtinen M, Dubin G, HPV PATRICIA Study Group (2012). Prevalence and risk factors for cervical HPV infection and abnormalities in young adult women at enrolment in the multinational PATRICIA trial. Gynecol Oncol.

[8] Dunne EF, Sternberg M, Markowitz LE, McQuillan G, Swan D, Patel S, Unger ER (2011). Human papillomavirus (HPV) 6, 11, 16, and 18 prevalence among females in the United States–National Health And Nutrition Examination Survey, 2003–2006: opportunity to measure HPV vaccine impact?. J Infect Dis.

[9] Oakeshott P, Aghaizu A, Reid F, Howell-Jones R, Hay PE, Sadiq ST, Lacey CJ, Beddows S, Soldan K (2012). Frequency and risk factors for prevalent, incident, and persistent genital carcinogenic human papillomavirus infection in sexually active women: community based cohort study. BMJ.

[10] Nielsen A, Kjaer SK, Munk C, Iftner T (2008). Type-specific HPV infection and multiple HPV types: prevalence and risk factor profile in nearly 12,000 younger and older Danish women. Sex Transm Dis.

[11] Delere Y, Remschmidt C, Leuschner J, Schuster M, Fesenfeld M, Schneider A, Wichmann O, Kaufmann AM (2014). Human Papillomavirus prevalence and probable first effects of vaccination in 20 to 25 year-old women in Germany: a population-based cross-sectional study via home-based self-sampling. BMC Infect Dis.

[12] Scheidt-Nave C, Kamtsiuris P, Gosswald A, Holling H, Lange M, Busch MA, Dahm S, Dolle R, Ellert U, Fuchs J, Hapke U, Heidemann C, Knopf H, Laussmann D, Mensink GB, Neuhauser H, Richter A, Sass AC, Rosario AS, Stolzenberg H, Thamm M, Kurth BM (2012). German health interview and examination survey for adults (DEGS) - design, objectives and implementation of the first data collection wave. BMC Public Health.

[13] Delere Y, Schuster M, Vartazarowa E, Hansel T, Hagemann I, Borchardt S, Perlitz H, Schneider A, Reiter S, Kaufmann AM (2011). Cervicovaginal self-sampling is a reliable method for determination of prevalence of human papillomavirus genotypes in women aged 20 to 30 years. J Clin Microbiol.

[14] Remschmidt C, Kaufmann AM, Hagemann I, Vartazarova E, Wichmann O, Delere Y (2013). Risk factors for cervical human papillomavirus infection and high-grade intraepithelial lesion in women aged 20 to 31 years in Germany. Int J Gynecol Cancer.

[15] Genesis Online Database Federal Statistical Office WNa;https://www-genesis.destatis.de/genesis/online

[16] Crochard A, Luyts D, di Nicola S, Goncalves MA (2009). Self-reported sexual debut and behavior in young adults aged 18–24 years in seven European countries: implications for HPV vaccination programs. Gynecol Oncol.

[17] Jensen KE, Munk C, Sparen P, Tryggvadottir L, Liaw KL, Dasbach E, Nygard M, Kjaer SK (2011). Women’s sexual behavior. Population-based study among 65,000 women from four Nordic countries before introduction of human papillomavirus vaccination. Acta Obstet Gynecol Scand.

[18] Bundeszentrale für gesundheitliche Aufklärung (BZgA): **Youth Sexuality. Repeat Survey of 14 to 17-year-olds and their parents– current focus: migration – 2010****.** http://publikationen.sexualaufklaerung.de/cgi-sub/fetch.php?id=687. Accessed on February 20, 2013. 2010

[19] : *Machs mit*. [http://www.machsmit.de/kampagne/]

[20] Franceschi S, Castellsague X, Dal Maso L, Smith JS, Plummer M, Ngelangel C, Chichareon S, Eluf-Neto J, Shah KV, Snijders PJ, Meijer CJ, Bosch FX, MuÃ±oz N (2002). Prevalence and determinants of human papillomavirus genital infection in men. Br J Cancer.

[21] Kjaer SK, Munk C, Winther JF, Jorgensen HO, Meijer CJ, van den Brule AJ (2005). Acquisition and persistence of human papillomavirus infection in younger men: a prospective follow-up study among Danish soldiers. Cancer Epidemiol Biomarkers Prev.

[22] Langille DB, Asbridge M, Flowerdew G, Allen M (2010). Associations of sexual risk-taking with having intercourse before 15 years in adolescent females in Cape Breton, Nova Scotia, Canada. Sex Health.

[23] Greenberg J, Magder L, Aral S (1992). Age at first coitus. A marker for risky sexual behavior in women. Sex Transm Dis.

[24] Olesen TB, Jensen KE, Nygard M, Tryggvadottir L, Sparen P, Hansen BT, Liaw KL, Kjaer SK (2012). Young age at first intercourse and risk-taking behaviours–a study of nearly 65 000 women in four Nordic countries. Eur J Public Health.

[25] Buston K, Williamson L, Hart G (2007). Young women under 16 years with experience of sexual intercourse: who becomes pregnant?. J Epidemiol Community Health.

[26] Giambi C, Donati S, Carozzi F, Salmaso S, Declich S, Atti ML, Ronco G, Alibrandi MP, Brezzi S, Collina N, Franchi D, Lattanzi A, Minna MC, Nannini R, Barretta E, Burroni E, Gillio-Tos A, Macallini V, Pierotti P, Bella A (2013). A cross-sectional study to estimate high-risk human papillomavirus prevalence and type distribution in Italian women aged 18–26 years. BMC Infect Dis.

[27] Schiffman M, Castle PE, Jeronimo J, Rodriguez AC, Wacholder S (2007). Human papillomavirus and cervical cancer. Lancet.

[28] Giambi C, Donati S, Declich S, Salmaso S, Degli Atti ML, Alibrandi MP, Brezzi S, Carozzi F, Collina N, Franchi D, Lattanzi A, Meda M, Minna MC, Nannini R, Scherillo I, Bella A, PreGio Working Group (2011). Estimated acceptance of HPV vaccination among Italian women aged 18–26 years. Vaccine.

[29] Donadiki EM, Jimenez-Garcia R, Hernandez-Barrera V, Carrasco-Garrido P, Lopez de Andres A, Velonakis EG (2012). Human papillomavirus vaccination coverage among Greek higher education female students and predictors of vaccine uptake. Vaccine.

[30] Blodt S, Holmberg C, Muller-Nordhorn J, Rieckmann N (2012). Human Papillomavirus awareness, knowledge and vaccine acceptance: a survey among 18–25 year old male and female vocational school students in Berlin, Germany. Eur J Public Health.

[31] Delere Y, Bohmer MM, Walter D, Wichmann O (2013). HPV vaccination coverage among women aged 18–20 years in Germany three years after recommendation of HPV vaccination for adolescent girls: results from a cross-sectional survey. Hum Vaccin Immunother.

[32] Sadry SA, De Souza LR, Yudin MH (2013). The impact of ethnicity on awareness and knowledge of and attitudes towards the human papillomavirus and vaccine among adult women. J Obstet Gynaecol Can.

[33] World Health Organization (2008). Preparing for the Introduction of HPV Vaccine in the WHO European Region. Strategy paper.

[34] Szarewski A, Cadman L, Mesher D, Austin J, Ashdown-Barr L, Edwards R, Lyons D, Walker J, Christison J, Frater A, Waller J (2011). HPV self-sampling as an alternative strategy in non-attenders for cervical screening - a randomised controlled trial. Br J Cancer.

[CR35] The pre-publication history for this paper can be accessed here:http://www.biomedcentral.com/1471-2458/14/1248/prepub

